# SAE3D: Set Abstraction Enhancement Network for 3D Object Detection Based Distance Features

**DOI:** 10.3390/s24010026

**Published:** 2023-12-20

**Authors:** Zheng Zhang, Zhiping Bao, Qing Tian, Zhuoyang Lyu

**Affiliations:** 1 School of Information, North China University of Technology, Beijing 100144, China; zhangzheng@ncut.edu.cn (Z.Z.); wiskey@mail.ncut.edu.cn (Z.B.); 2 School of Information, Brown University Computer Science and Applied Math, Providence, RI 02912, USA; zhuoyang_lyu@brown.edu

**Keywords:** 3D object detection, distance features, SA layer enhancement

## Abstract

With the increasing demand from unmanned driving and robotics, more attention has been paid to point-cloud-based 3D object accurate detection technology. However, due to the sparseness and irregularity of the point cloud, the most critical problem is how to utilize the relevant features more efficiently. In this paper, we proposed a point-based object detection enhancement network to improve the detection accuracy in the 3D scenes understanding based on the distance features. Firstly, the distance features are extracted from the raw point sets and fused with the raw features regarding reflectivity of the point cloud to maximize the use of information in the point cloud. Secondly, we enhanced the distance features and raw features, which we collectively refer to as self-features of the key points, in set abstraction (SA) layers with the self-attention mechanism, so that the foreground points can be better distinguished from the background points. Finally, we revised the group aggregation module in SA layers to enhance the feature aggregation effect of key points. We conducted experiments on the KITTI dataset and nuScenes dataset and the results show that the enhancement method proposed in this paper has excellent performance.

## 1. Introduction

With the development of unmanned driving and other technologies, understanding 3D scenes based on the point cloud has become a popular research topic. Compared to traditional images, point cloud data have unique advantages. The strong penetration of LiDAR makes the point cloud less susceptible to external factors such as weather and light. However, point clouds are also characterized by sparseness and disorder, and the reflectivity of LiDAR decreases as the measurement distance increases. This leads to poor characterization of objects at a distance, causing a drop in detection accuracy. How to deal with these characteristics of point clouds has become the key to improving accuracy in 3D detection tasks.

**Figure 1:**
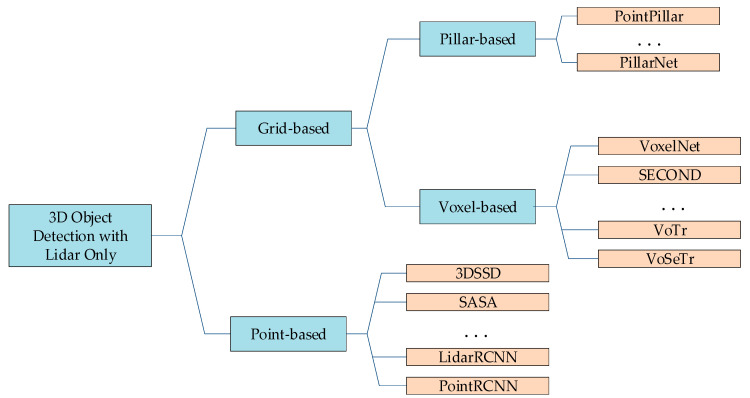
Overview of related work.

In recent years, to efficiently utilize the information provided by the point cloud, researchers have proposed a number of schemes, as shown in Figure 1. These are mainly divided into two types based on different processing methods:

(a) Grid-based methods, which partition the sparse points into regular voxel or pillar grids, and process them through 3D or 2D convolutional networks.
(b) Point-based methods, which directly perform feature learning on point sets with SA which are most often utilized to sample the key points and aggregate features.

Compared to the point-based methods, the grid-based methods increase the computational speed of network inference, but also cause information loss during the voxelization. Therefore, to ensure the full utilization of information in point sets, a point-based methods enhancement network is proposed in this paper.

The core of the point-based 3D object detection methods is the SA layer, which was first proposed by Qi et al. [1]. In prior research, the SA layer has been revised using many methodologies, and how to fully utilize the information of each point and reduce inference time has become a priority in point-based methods. In 3DSSD [2], to speed up the inference, researchers first adopted a 3D single stage object detector and proposed a feature-based farthest point sample module (F-FPS). This module utilizes the feature information of the point sets to sample key points in order to maintain adequate interior points of different foreground instances. SASA [3] uses a semantic-segmentation-based farthest point sample module (S-FPS), which utilizes point cloud features distinguish the foreground points from the background points through a small semantic segmentation module to better access key points. However, the point cloud features used by these algorithms only utilize the raw features of the point cloud, i.e., the reflectivity and 3D coordinates that reveal spatial information of each point in the point cloud, and distance characteristics are not taken into consideration. In the actual measurement, due to the attenuation characteristics of LiDAR and the limitation of the observation angle, the reflectivity of the measured point decreases as the object moves further away, and the projection of the object in the point cloud also decreases.

Therefore, based on the distance characteristics related to the point cloud, we propose three feature enhancement modules to more efficiently utilize the semantic information contained within the point cloud. Firstly, we propose the initial feature fusion module, in which the distance feature is extracted from the point cloud and incorporated with the raw features of each point. Secondly, we introduce a key point feature enhancement module. During the group aggregation in SA, the self-characterization of the key points will be weakened, but it is crucial for distinguishing whether the key point is a foreground or background point. Therefore, after each sampling aggregation, the multi-attention mechanism is used to strengthen the features of key points and fuse them with the aggregated features. Finally, to enhance the effect of group aggregation in SA, we revised the original grouping module. In the original module, multiple points nearest to the key points are taken to participate in feature aggregation after sampling over the key points. However, only the spatial location is considered, which may result in features belonging to different categories being mixed together during the aggregation process. This leads to a decrease in the performance of the semantic segmentation module before S-FPS, which in turn degrades the sampling effect of S-FPS. Therefore, we optimize the grouping module by selecting the points with the closest features as the aggregation points from among multiple points closest to the key points.

In summary, the main contributions of this article are summarized as follows:

- We propose a key points self-features enhancement module to enhance the self-features of the key points. In this module, we introduce the multi-attention mechanisms to enhance the raw features and distance features to retain the semantic information of the key points as much as possible during each SA layer.
- We propose an initial feature fusion module to extract the distance features of the point cloud and fuse the distance features into the raw features of the point sets. This module makes the features of the distant points more significant and thus improves the detection accuracy of the distant instances.
- We revise the group aggregation module in the set abstraction. We make a second selection after the first selection of points within a fixed distance around the key point. In second selection, we take the features into account to enhance the sampling effect of S-FPS.

## 2. Related Work

Since the growing data on point clouds bring huge challenges to existing point cloud processing networks, it is important to compress the point cloud before processing it. Different compression algorithms used may affect the subsequent detection effect. For example, Sun X et al. [4] optimized the processing of large-scale point cloud data and their algorithm [5] further streamlines the network for point cloud processing. The algorithm [6] makes the spatial distribution of the compressed point cloud more similar to the original point cloud, which is very useful for subsequent point cloud processing.

The point cloud compression algorithms mentioned above play a significant role in the point cloud detection algorithms we will discuss next.

### 2.1. Grid-Based Methods

Grid-based methods are mainly divided into two categories: voxel-based methods and pillar-based methods. In voxel-based methods, an irregular point cloud is first con-verted into regular voxels, which are then fed into the network. Voxel-Net [7] is a pioneering network that converts point cloud into voxels, and then utilizes 3D convolutional networks to predict 3D bounding boxes. Yan et al. [8] proposed 3D sparse convolution, which reduces the computation of traditional 3D convolution and greatly improves the detection efficiency of voxel-based detection networks. Voxel-Transformer [9] and Voxel Set Transformer [10] introduce modules such as Transformer [11] and Set Transformer [12], respectively, on the basis of voxels to improve the detection accuracy. SFSS-Net [13] is a unique algorithm to filter background points before the voxelization to reduce computational complexity. Pillar-based methods such as Point Pillars [14] divide the space into regular pillars, which are compressed and then fed into a 2D convolutional network, greatly increasing the network inference speed. Pillar Net [15] uses a sparse convolutional-based encoder network for spatial feature learning, and the Neck module for high-level and low-level feature fusion to improve the accuracy of pillar-based detection methods. Pillar Next [16] first compares different local point aggregators (pillar, voxel and multi-view fusion) from the perspective of computational budget allocation. Research shows that pillars can achieve better performance compared to voxels. Grid-based methods lose more semantic information in the process of converting an irregular point cloud into regular voxels or pillars. This may lead to poor performance in the final detection accuracy.

### 2.2. Point-Based Methods

Point-based methods generally perform feature extraction directly on the point sets. This approach obtains key points and aggregates points around them by means of sampling and group aggregation for feature extraction. Point-based methods were first proposed by Qi et al. [17] and later improved and refined by Qi et al. [1]. Shi et al. [18] first proposed to extract the foreground points by segmentation and utilize the features of these points for the bounding box regression to improve the detection accuracy. Yang et al. [2] utilized one-stage detection to improve the inference speed and proposed the F-FPS, to make the sampled key points closer to the foreground instances. SASA [3] is used to predict scores of each point by a small semantic segmentation module to make abstracted point sets focus on object areas. Chen et al. [19] introduced density information from point clouds using the Multilayer Perceptron (MLP) and integrated it with features extracted by grouping operations in the point-based method.

Since point-based 3D object detection is directly processing the point sets, point-based methods result in high computational consumption and long network inference time. However, relative to the voxel-based methods, point-based methods can maximize the retention of the semantic information of the point cloud and achieve higher detection accuracy. Therefore, this paper adopts the point-based object detection network and aims to utilize the original information of the point cloud more efficiently.

## 3. Proposed Methods

In this section, we will introduce in detail the network architecture of the SAE3D proposed in this paper. This enhancement network consists of three main parts: an initial feature fusion module, a key points self-features enhancement (KSFE) module and a revised group aggregation (RGA) module.

**Figure 2:**
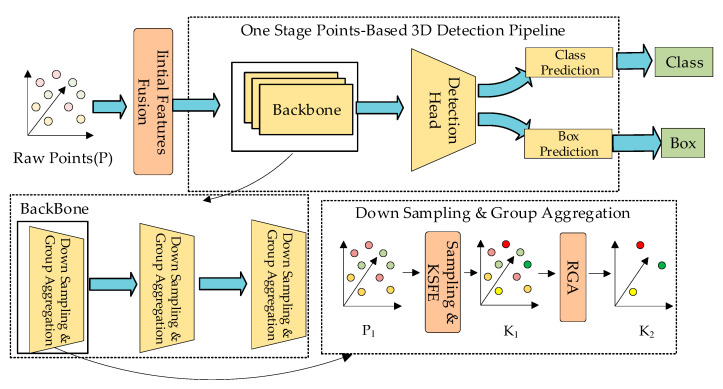
Overall flowchart. The raw point cloud *P* goes through the initial feature fusion module to get *P*_1_, *P*_1_ is input to the backbone, backbone consists of three SA (set abstraction) layers. *P*_1_ is first put through the down sampling and then through the KSFE (key points self-feature enhancement module) to get *K*_1_, and finally through the RGA (revised group aggregation module) to get *K*_2_.

As shown in Figure 2, the overall architecture is a one-stage point-based 3D object detection network. Firstly, we define the raw points fed into the network as *P*; the initial feature fusion module extracts the distance features and integrates initial features of each point in *P*. After integration, we feed *P* into the backbone, which contains three SA layers, and we refer to the input of each SA layer as *P*_1_. In SA layers, we first sample the key points *K* from *P*_1_, and then we feed *K* into the key points feature enhancement module to enhance the self-features of *K*. After enhancement, we obtain *K*_1_. Finally, the revised group aggregation module is used to aggregate the points around *K*_1_ to obtain the aggregated key points *K*_2_. *K*_2_ is the final output of each SA layer.

After the backbone is complete, to improve the prediction accuracy, this paper adopts the bounding box prediction mechanism in Vote-Net [20] to predict the bounding box similarly to SASA [3].

We will explain each module in detail below.

### 3.1. Initial Features Fusion Module

Before the SA layers, we utilize the initial features fusion module to extract the distance features and integrate these with features of the raw point sets. The relevant features of the raw point cloud are very sensitive to the measurement distance. In the actual measurement, as the distance increases, the reflectivity of LiDAR decreases, which leads to the problem that the features of the long-distance points are not obvious and thus reduce the detection accuracy. Therefore, we believe that distance features are very important for improving target detection accuracy.

#### 3.1.1. Distance Features

Traditional algorithms often involve calculations such as squares and roots when calculating distance. This costs a lot of computational resources if we directly let distance represent the distance feature of each point in the point clouds. Therefore, our distance feature is defined as follows:

(1) $$DF_{p}=\frac{\left|x_{p}\right|+\left|y_{p}\right|+\left|z_{p}\right|}{Scale}\;\;\;\;\;\;\;\;\;\;\;\;\;\;\;\;\;\;\;p\in P$$

where *P* is the raw point set, *DF_p_* and (*x_p_*, *y_p_*, *z_p_*) represent the distance feature and the coordinates of the *p*, respectively. *Scale* is the scaling factor. We utilize the sum of absolute values of the three-axis coordinates of *p* to represent the distance of a point. The *Scale* will be set in the experiment.

#### 3.1.2. Feature Fusion

**Figure 3:**
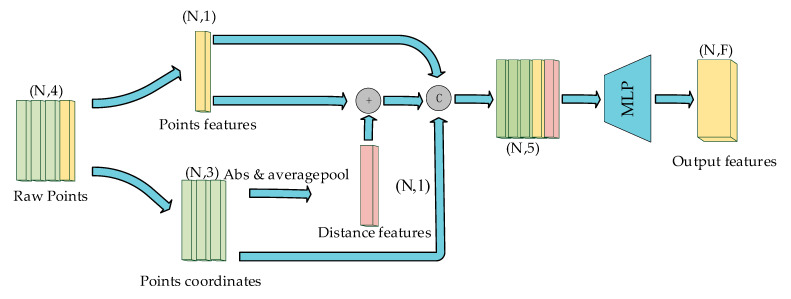
Initial features fusion module (where *N* stands for the number of input point clouds and *F* stands for the number of feature layers for each point in the output. “*C*” represents the stitching operation and “*+*” represents the numerical summing operation.).

We process the initial feature fusion as shown in Figure 3. Since the reflectivity of each point decreases with the increase of the measurement distance, we adopt the approach of adding distance features with the initial features of the point cloud to strengthen the representation of long-distance points. Finally, we perform fusion operations on the coordinates and related features of the point cloud through the splicing operation. However, these features have not been processed enough, so we use the Multilayer Perceptron (MLP) to further extract the depth features.

### 3.2. Key Points Features Enhancement Module

**Figure 4:**
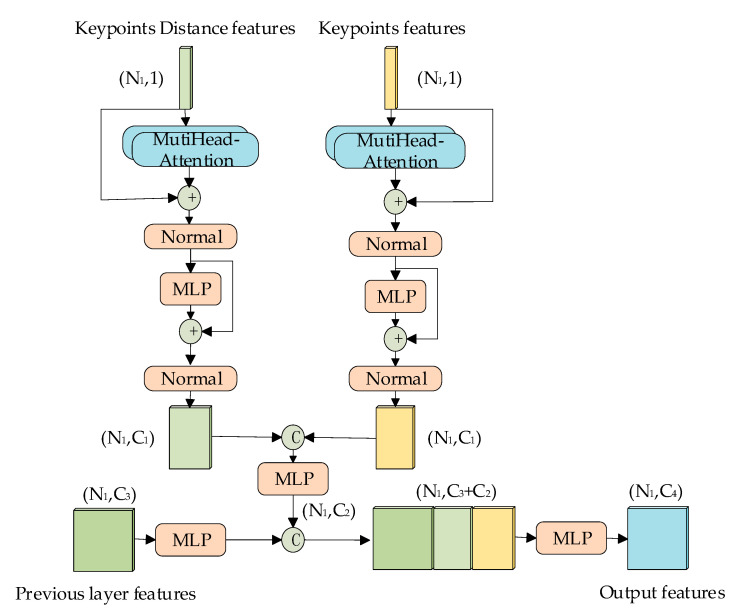
Key points self-features enhancement module (where *N*_1_ is the number of key points after sampling, and *C_i_* is the number of feature channels in each stage. “C” stands for the stitching operation and “+” stands for the numerical summing operation.).

In SA layers, the key points obtained from the sampling will undergo feature aggregation with the surrounding points, and the self-features of the key points will be diminished after aggregation with max or average pooling. However, each key point has its own unique features in the point cloud data, and these features contain important information included where the key point is located in the point cloud and what kind of object the key point represents. However, the feature aggregation will cause the loss of such information. Therefore, we propose a key points self-feature enhancement module as shown in Figure 4, which enhances the distance features and raw features of the key points, integrating them into the aggregated features.

#### Feature Enhancement Module

In order to make the self-features of the key points distinctive, we adopt the multi-attention mechanism to enhance the distance features and raw features of the key points. The features are strengthened through the multi-head self-attention mechanism; the self-attention algorithm essentially uses matrix multiplication to calculate the relationship between each patch and the other patches in the query. The specific formulas are as follows:

(2) $$Attention(Q,K,V)=Softmax(\frac{QK^{T}}{\sqrt{d_{k}}})V$$

(3) $$Q=F\times W_{q}$$

(4) $$K=F\times W_{k}$$

(5) $$V=F\times W_{v}$$

where *F* is the self-features of the key points, *W_q_*, *W_k_* and *W_v_* are the learnable weight matrices. Equations (3)–(5) represent that *F* obtains *Q*, *K*, and *V* through three separate MLPs. After obtaining *Q*, *K*, and *V*, we use Equation (2) to finally obtain the attention features. After that we employ the splicing method to combine them together. Finally, we carry out the integration of the aggregated features of the key points with their self-features using the MLP to accomplish the enhancement of self-features of key points.

### 3.3. Revised Group Aggregation Module

In the process of sampling key points, we follow the S-FPS and D-FPS combined sampling strategy, which is similar to that of SASA [3]. A small semantic segmentation module is adopted in the network structure to compute the classification score for each point to distinguish between foreground and background points in the point cloud. The input features to the segmentation network are those obtained from grouping of the point sets. In the general grouping operation, the selection of points used for aggregation around the key points only considers the spatial location from the key points, not taking into account the feature distance from the key points. In this paper, it is argued that this aggregation operation diminishes the borderlines of the different instances and reduces the effectiveness of the segmentation module in predicting the classification scores of each point in the network, thus affecting the sampling performance of S-FPS. To avoid these problems, we perform a second selection after selecting points within a certain distance from each key point. In the second selection, we introduce the feature distance to ensure that the features of the selected points are similar to those of the key point. By doing so, we can enhance the performance of the segmentation in this network.

#### Group Aggregation Method

**Figure 5:**
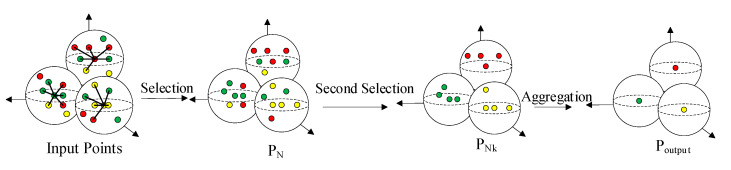
Revised group aggregation module (points with similar colors in the figure represent similar features, *P_N_* is the point obtained by the first selection around the key point, *P_Nk_* is the point obtained by the second selection, and *P_output_* is the final point output after group aggregation).

The particular operation is shown in Figure 5. First, we select *N* points as a point set *P_N_* within the sphere with radius *R* around the key point, and calculate the feature distance *D_f_* between the points and the key points, which we define as follows:

(6) $$D_{f}={\left|f_{keypo\mathrm{int}s}-f_{n}\right|}_{}\;\;\;\;\;\;\;\;\;\;\;\;\;\;\;n\in N$$

where *f_keypoints_* and *f_n_* separately represent the features of the key points and the features of the points around the key points. Before calculation, these features will go through a simple MLP to ensure that the features channel is one-dimensional. After obtaining *D_f_*, we select the *N_k_* points with the smallest *D_fk_* (*k* = 1, 2, …, *N_k_*) in *P_N_* as a point set *P_Nk_*. The *P_Nk_* will be used for subsequent features aggregation. In this way, we further strengthen the semantic information of the key points. This can help S-FPS to better distinguish the foreground points from the background points before sampling.

### 3.4. Prediction Head

The overall architecture in this paper consists of three SA layers with a bounding box prediction network. Similarly, our bounding box prediction network adopts the bounding box prediction mechanism from Vote-Net [20]. The voting point indicating the center of mass of the corresponding object is first computed from the candidate point features, and then the points in the vicinity of each voting point are aggregated to estimate the bounding box of the detected target.

### 3.5. Loss

The loss function in SAE3D is inherited from SASA [3]. The overall loss function is expressed as follows:

(7) $$L=L_{v}+L_{c}+L_{r}+L_{seg}$$

where *L_c_* and *L_r_* are the losses for the classification and regression, *L_v_* is the loss generated when calculating the vote in the point voting head proposed in Vote-Net [20]. *L_seg_* is the total segmentation loss proposed in SASA [3].

*L_c_* and *L_r_* are the traditional losses for object detection. They can help the network better predict the bounding box and classification of the object to be detected. *L_v_* mainly serves to predict the center point of the object to improve the accuracy of bounding box prediction. *L_seg_* mainly serves to perform semantic segmentation before the S-FPS to better differentiate between foreground and background points. This can improve the sampling capability of the S-FPS. Therefore, we adopt these loss functions to better train our model.

## 4. Experiment

### 4.1. Datasets

The network we proposed is validated on the KITTI dataset and nuScenes dataset.

#### 4.1.1. KITTI Dataset [21]

The KITTI dataset is a widely used public dataset in the field of computer vision, which is primarily utilized to study and evaluate tasks such as autonomous driving, scene understanding, and target detection. The dataset is based on the streets of Karlsruhe, Germany, and comprises a wide range of urban driving scenarios. The KITTI dataset has become the mainstream standard for 3D object detection in traffic scenes due to its provision of data from real-world scenarios with a high level of realism and representative value.

In the original KITTI dataset, each sample comprises multiple consecutive frames of point cloud data. In our experiment, a total of 7481 point clouds are included along with 3D bounding boxes for training purposes, and 7581 samples are allocated for testing. We adopt a general setup where the training samples are further subdivided into 3712 training samples and 3769 testing samples. Our experimental network is trained on the training samples and validated on the testing samples.

#### 4.1.2. NuScenes Dataset [22]

The nuScenes dataset is one of the more challenging datasets for autopilot research. It comprises 380,000 LiDAR scans from 1000 scenes and is labeled with up to 10 object categories, including 3D bounding boxes, object velocities, and attributes. The detection range is 360 degrees. The nuScenes dataset is evaluated using metrics such as the commonly used mean Average Precision (mAP) and the novel nuScenes Detection Score (NDS), which reflects the overall quality of measurements across multiple domains.

When transferring the nuScenes dataset, we combine LiDAR points from the current key frame and previous frames within 0.5 s, which involves up to 400 k LiDAR points in a single training sample. We then reduce the number of input LiDAR points. Specifically, we voxelize the point cloud from the key frame as well as the stacked previous frames with pixel sizes of (0.1 m, 0.1 m, 0.1 m), then randomly select 16,384 and 49,152 voxels from the key frame and the previous frames, respectively. For each selected voxel, we randomly select one internal LiDAR point. A total of 65,536 points were fed into the network with 3D coordinates, reflectivity, and timestamps.

#### 4.1.3. Evaluation Indicators

In the experiment on the KITTI dataset, two precision metrics are used. One is the 11-point interpolated average precision (*AP*) proposed by Gerard et al. [23], and the other is the average precision *AP*|*_R_*_40_ for 40 recalled positions proposed by Simonelli et al. [24]. The Intersection over Union (IoU) threshold for all precision calculations is 0.70. The specific formulas of *AP*|*_R_* are as follows:

(8) $$AP|R=\frac{1}{\left|R\right|}\sum_{r\in R}\rho_{interp}(r)$$

(9) $$\rho_{interp}(r)=\underset{r’:r’\geq r}{\max}\;p(r’)$$

where *p*(*r*) gives the precision at recall *r. AP* applies exactly 11 equally spaced recall levels: *R*_11_ = {0, 0.1, 0.2, …, 1} and *AP*|*_R_*_40_ applies recall levels: *R*_40_ = {1/40, 2/40, 3/40, …, 1}. We mainly use *AP* as an accuracy indicator and *AP*|*_R_*_40_ will be applied in the ablation experiment in Section 4.5.

In the nuScenes dataset, as mentioned above, we apply the *NDS* and *mAP* as the evaluation indicator. The specific formulas for *NDS* are expressed as follows:

(10) $$NDS=\frac{1}{10}\left[5mAP+\sum_{mTP\in TP}\left(1-\min(1,mTP)\right)\right]$$

where *mTP* is the mean True Positive metrics and consists of 5 metrics: average translation error, average scale error, average orientation error, average velocity error, and average attribute error.

### 4.2. Experimental Setting

SAE3D is implemented based on the Appended [25] and is trained on a single GPU. All experiments were performed on Ubuntu 16.04 and NVIDIA RTX-2080Ti.

#### 4.2.1. Setting in KITTI Dataset

During the training process, the batch size takes the value of 2, and 16,384 points are randomly selected from the remaining points in each batch to input into the detector. In terms of network parameters, the number of key points in the three SA layers is set to 4096, 1024, and 512, respectively, and the scaling factor Scale for the distance feature takes the value of 120.

Adam optimizer [26] and a periodically varying learning rate were adopted in the training for a total 80 epochs, with the initial value of the learning rate set to 0.001. Additionally, we used three commonly used data augmentation methods during training: randomly flipping the X-axis with respect to the Y-axis, random scaling, and randomly rotating the Z-axis.

#### 4.2.2. Setting in nuScenes Dataset

During the training process, the batch size takes the value of 1. Adam optimizer and a periodically varying learning rate were adopted in the training for a total of 10 epochs, with the initial value of the learning rate set to 0.001.

To handle the huge number of points in the nuScenes dataset, four SA layers are adopted. The number of key points in these four SA layers is set to 16,384, 4096, 3072, and 2048, respectively.

### 4.3. Results

The detection performance of the SAE3D model is evaluated on the KITTI dataset and nuScenes dataset against some existing methods proposed in the literature.

In the KITTI dataset, the test set is categorized into three levels of difficulty, i.e., “Easy”, “Moderate”, and “Hard”, based on the difficulty of detection. We take the 3D bounding box average precision (3D AP) of the “Car” category as the main evaluation, as this is usually adopted as the main indicator in KITTI datasets. As shown in Table 1, compared with the baseline network SASA, 3D AP is improved by 0.544% and 0.648% in the difficulty levels of “Moderate” and “Hard”, respectively. The detailed precision improvements will be shown in Section 4.5.

In the nuScenes dataset, as shown in Table 2, compared with the baseline network, SAE3D achieved 3.3% and 1.7% improvement in the indicators of NDS and mAP, respectively.

### 4.4. Enhancement Validation

To verify the enhancement effect of the proposed network in this paper, we utilize SASA [3] and Point-RCNN [18] as two baseline networks for testing in the KITTI dataset. Both baseline networks are point-based 3D object detection networks, where SASA [3] is a one-stage object detection network and Point-RCNN [18] is a two-stage object detection network. The experiments introduce the enhanced network proposed in this paper into both of these networks, effectively improving the detection performance of the original benchmark network.

Table 3 shows the improvement in the accuracy of the 3D detection frames of the “Car” category in the enhanced networks of SASA [3] and Point-RCNN [18], respectively.

After the introduction of the enhanced network in SASA [3], the 3D AP of the “Car” decreases slightly in the “Easy” difficulty, but increases by 0.544% and 0.648% in the “Moderate” and “Hard” difficulties, respectively.

After introducing the enhanced network in Point-RCNN [18], the accuracy of the 3D AP is improved by 0.137%, 0.593%, and 0.885% in “Easy”, “Moderate”, and “Hard” difficulties, respectively.

### 4.5. Ablation Experiment

In this paper, ablation experiments are designed to verify the actual effect of each module. All modules are trained on the training set of the KITTI dataset and evaluated on the validation set for the “Car” category of the KITTI dataset.

In this section we added BBox AP, BEV AP, and AOS AP alongside 3D AP as the evaluation indicator. BBox AP represents the average precision of the 2D bounding box, while BEV AP denotes the average precision of the detection boxes in bird’s-eye view. These two indicators provide detection box accuracy from different perspectives, aiding in a better understanding of the spatial precision of the detection boxes predicted by our model. AOS AP stands for the average precision of the detected target’s rotation angle, indicating the accuracy of the object orientation predicted by our model.

#### 4.5.1. Initial Feature Fusion Module

As shown in Table 4, the initial feature fusion module proposed in this paper is of great help to improve the precision of 3D bounding box. The improvement of this module is most evident in the difficulty levels of “Moderate” and “Hard”. Compared to the baseline network used in this paper, in the “Moderate” and “Hard” difficulty levels, the 3D bounding box accuracy improvement of this module is 0.551% and 0.811%, respectively. Additionally, the improvement in 2D bounding box accuracy is 0.186% and 0.811%, while the bounding box accuracy improvement in BEV view is 0.257% and 1.048%, respectively.

As shown in Table 5, when using the AP|*_R_*_40_, the improvement in the accuracy of the 3D bounding box is 2.549% and 2.582% for the difficulties of “Moderate” and “Hard”, respectively. The improvement in the accuracy of 2D bounding box is 1.976% and 0.533%, respectively, and the improvement in the accuracy of bounding box in BEV view is 0.295% and 2.257%, respectively.

#### 4.5.2. Key Points Self-Features Enhancement Module

As shown in Table 4, this module improves the detection accuracy of the 3D bounding box and the accuracy of bounding box detection in BEV view. The detection accuracy of the 3D bounding box is improved by 0.339% and 0.746% under the difficulty levels of “Moderate” and “Hard”, respectively, and the detection accuracy of the bounding box in BEV view is improved by 0.118%, 0.565%, and 1.349% in “Easy”, “Moderate”, and “Hard” levels of difficulty, respectively.

As shown in Table 5, the accuracy of the 3D bounding box is improved by 2.362% and 2.467% for the “Moderate” and “Hard” levels of difficulty, respectively, when using AP|*_R40_*. The accuracy of the bounding box in BEV view is improved by 1.778%, 1.906% and 2.367% for “Easy”, “Moderate”, and “Hard” levels of difficulty, respectively.

#### 4.5.3. Revised Group Aggregation Module

As shown in Table 4, the detection accuracy of this module on BBOX is improved by 0.316% and 0.376% under the difficulty levels of “Moderate” and “Hard”, respectively. Additionally, compared with the baseline network, the module improves other metrics such as 3D bounding box and steering angle accuracies.

As shown in Table 5, when AP|*_R40_* is used, the detection accuracy improvement on BBOX is 2.051% and 0.555% at “Moderate” and “Hard” levels, respectively.

### 4.6. Detection Effect

**Figure 6:**
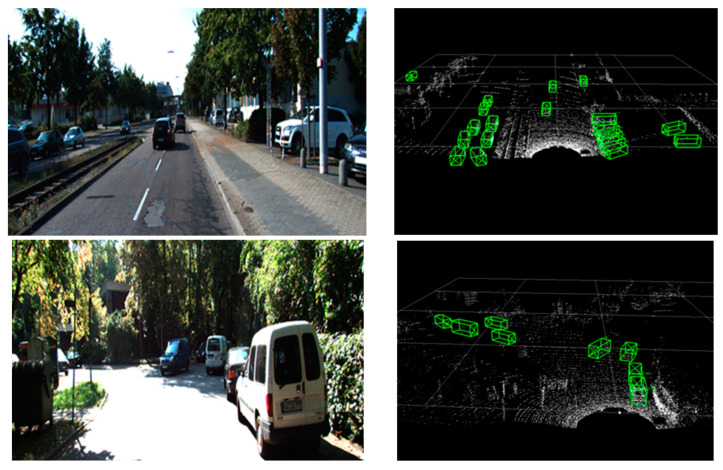
Actual detection effect diagram in KITTI dataset (left are the pictures of the real scenes, right are the detection 3D bounding boxes predicted in the point cloud).

Figure 6 shows the actual detection effect. Although there is still a small part of the missed detection problem, most of the vehicles are detected and the accuracy of the 3D bounding box is high.

## 5. Discussion

In this paper, we continue to explore the possibilities of the point-based 3D object detection. Point cloud data are vast and contains a wealth of information, both useful and redundant. We believe that there is still underutilized information within the point cloud. Therefore, we proposed the SAE3D. The results demonstrate that extracting more useful information and enhancing the relevant information in the point cloud can improve the final detection accuracy.

## 6. Conclusions

In this paper, we proposed SAE3D with three enhancement modules: an initial feature fusion module, a key points self-feature enhancement module, and a revised group aggregation module. We provide a detailed description of the design ideas and implementation of these modules in this paper. We conducted testing using the KITTI and nuScenes datasets and designed ablation experiments on the KITTI dataset to analyze the enhancement of each module in detail. The results demonstrate that all three enhancement modules we propose contribute to enhancing detection accuracy. Our SAE3D suggests that there are still useful characteristics in point clouds that are not fully utilized, and some of them can assist in extracting information from the point clouds more effectively. We believe that exploring additional potential characteristics of point clouds can further enhance 3D scene understanding.

## Figures and Tables

**Table 1:** The detection results of 3D AP for “Car” in KITTI.

Methods	Car 3D AP (%)		
	Easy	Moderate	Hard
**SECOND** [8]	84.656	75.966	68.712
**Voxel Net** [7]	77.478	65.119	57.736
**Point Pillars** [14]	82.588	74.317	68.995
**Point-RCNN** [18]	89.023	78.246	77.554
**Vox Set Tran** [10]	88.869	78.766	77.576
**SASA** [3]	**89.108**	78.847	77.588
**SAE3D**	89.059	**79.391**	**78.236**

**Table 2:** Results from the nuScenes validation set. Evaluation metrics include NDS, mAP, and 10 classes. Abbreviations: pedestrian (PED.), traffic cone (T.C.), construction vehicle (C.V.).

Methods	NDS	mAP	Car	Truck	Bus	Trailer	C.V.	Ped.	Motor	Bicycle	T.C.	Barrier
**Point Pillars** [14]	45.2	25.8	70.3	32.9	44.9	18.5	4.2	46.8	14.8	0.6	7.5	21.3
**3DSSD** [2]	51.7	34.5	75.9	34.7	60.7	21.4	10.6	59.2	25.5	7.4	14.8	25.5
**SASA** [3]	55.3	36.1	71.7	42.2	63.5	29.6	12.5	62.6	27.5	9.1	12.2	30.4
**SAE3D**	**58.6**	**37.8**	72.4	44.1	62.7	31.2	15.9	60.4	30.1	12.8	10.1	31.6

**Table 3:** Enhancement effectiveness. Abbreviations: Distance features-based enhancement network proposed in this paper (SAE3D).

Methods	Car 3D AP (%)		
	Easy	Moderate	Hard
**SASA** [3]	89.108	78.847	77.588
**SASA** [3] **+ SAE3D**	89.059	79.391	78.236
**Improvement**	−0.049	+0.544	+0.648
**Point-RCNN** [18]	89.023	78.246	77.554
**Point-RCNN** [18] **+ SAE3D**	89.160	78.839	78.439
**Improvement**	+0.137	+0.593	+0.885

**Table 4:** Comparison table of the general accuracy enhancement effect of different modules. Abbreviations: initial feature fusion module (I), KSFE module (K), and RGA module (F).

+I	+K	+F	Car 3D AP (%)			Car BBOX AP (%)			Car BEV AP (%)			Car AOS AP (%)		
			Easy	Mod	Hard	Easy	Mod	Hard	Easy	Mod	Hard	Easy	Mod	Hard
			89.108	78.847	77.588	96.742	89.855	89.036	90.199	87.855	85.993	96.71	89.75	88.88
	√		88.971	79.246	78.334	96.473	89.847	89.163	**90.317**	**88.420**	**87.342**	96.44	89.81	89.07
		√	89.213	79.324	78.114	**96.813**	**90.171**	**89.412**	89.876	89.397	86.976	96.54	90.08	89.11
√			**89.167**	**79.398**	**78.399**	96.668	90.041	89.287	90.149	88.112	87.041	96.64	89.98	89.12
√	√	√	89.059	79.391	78.236	96.758	90.169	89.382	89.978	88.382	86.824	**96.71**	**90.10**	**89.22**

**Table 5:** Comparison table of the AP|*_R_*_40_ enhancement effect of different modules. Abbreviations: initial feature fusion module (I), KSFE module (K), and RGA module (F).

+I	+K	+F	Car 3D AP R40 (%)			Car BBOX AP R40(%)			Car BEV AP R40(%)			Car AOS AP R40(%)		
			Easy	Mod	Hard	Easy	Mod	Hard	Easy	Mod	Hard	Easy	Mod	Hard
			**91.592**	80.705	77.902	**98.289**	92.972	92.104	93.277	89.128	86.465	**98.26**	92.85	91.92
	√		91.457	83.067	80.369	98.111	94.583	92.469	**95.055**	**91.034**	**88.832**	98.09	94.52	92.35
		√	91.432	82.913	78.956	98.023	95.023	92.659	93.124	89.223	88.624	98.21	94.89	92.39
√			91.555	**83.254**	**80.484**	98.097	94.948	**92.637**	93.197	89.423	88.722	98.08	94.85	**92.44**
√	√	√	91.426	83.236	80.191	98.266	**95.036**	92.616	93.014	90.902	88.525	98.23	**94.93**	92.42

## Data Availability

Data are contained within the article.
